# Dioxin-like compounds and bone quality in Cree women of Eastern James Bay (Canada): a cross-sectional study

**DOI:** 10.1186/1476-069X-12-54

**Published:** 2013-07-02

**Authors:** Alexandra-Cristina Paunescu, Éric Dewailly, Sylvie Dodin, Evert Nieboer, Pierre Ayotte

**Affiliations:** 1 Axe santé des populations et pratiques optimales en santé. Centre de recherche du CHU de Québec, 2875, boulevard Laurier, Édifice Delta 2, bureau 600, Québec, QC G1V 2M2, Canada; 2 Laboratoire de toxicologie, INSPQ, 945, avenue Wolfe, Québec, QC G1V 5B3, Canada; 3 Axe santé des populations et pratiques optimales en santé. Centre de Recherche du CHU de Québec, Hôpital Saint-François-d'Assise, 10, rue de l’Espinay, Québec, QC G1L 3L5, Canada; 4 Department of Biochemistry and Biomedical Sciences, McMaster University, 1280 Main Street West, Hamilton, ON L8S 4K1, Canada

**Keywords:** Calcaneal Ultrasound Parameters, Dioxin-Like Compounds, Polychlorinated Biphenyls, Cree Women, Canada

## Abstract

**Background:**

Aboriginal populations living in Canada’s northern regions are exposed to a number of persistent organic pollutants through their traditional diet which includes substantial amounts of predator fish species. Exposure to dioxin-like compounds (DLCs) can cause a variety of toxic effects including adverse effects on bone tissue. This descriptive cross-sectional study was conducted to investigate the relationship between plasma concentrations of DLCs and bone quality parameters in Cree women of Eastern James Bay (Canada).

**Methods:**

Two hundred and forty-nine Cree women from seven communities in Eastern James Bay (Canada), aged 35 to 74 years old, participated in the study. In order to determine the total DLC concentration in plasma samples of participants, we measured the aryl hydrocarbon receptor-mediated transcriptional activity elicited by plasma sample extracts using a luciferase reporter gene assay. Plasma concentrations of mono-ortho-substituted dioxin-like polychlorinated biphenyls (DL-PCBs) 105, 118 and 156 were measured by gas chromatography–mass spectrometry. Bone quality parameters (speed of sound, m/s; broadband ultrasound attenuation, dB/MHz; stiffness index, %) were assessed by quantitative ultrasound at the right calcaneus with the Achilles InSight system*.* Several factors known to be associated with osteoporosis were documented by questionnaire. Multiple linear regression models were constructed for the three ultrasound parameters.

**Results:**

DL-PCBs 105 and 118 concentrations, but not the global DLC concentration, were inversely associated with the stiffness index, even after adjusting for several confounding factors. The stiffness index (log) decreased by −0.22% (p=0.0414) and −0.04% (p=0.0483) with an increase of one μg/L in plasma concentrations of DL-PCB 105 and DL-PCB 118, respectively. Other factors, including age, height, smoking status, menopausal status and the percentage of omega-6 polyunsaturated fatty acids (PUFAs) in erythrocyte membranes were negatively associated with one of the ultrasound parameters, while the percentage of omega-3 PUFAs in these membranes and levels of physical activity and education were positively associated with them.

**Conclusions:**

Our results show that an increase in plasma concentrations of DL-PCBs 105 and 118 was negatively associated with stiffness index, a measure of bone quality/strength, in women of this population. In addition to environmental contaminants, future studies should also consider PUFA intake as a factor influencing bone quality.

## Background

Osteoporosis is “a systemic skeletal disease characterized by low bone mass and micro-architectural deterioration of bone tissue, with a consequent increase in bone fragility and susceptibility to fractures” [1]. It is a multifactorial polygenic disease in which genetic determinants are modulated by hormonal, environmental, and nutritional factors [2], is asymptomatic and progress silently with age [3]. Eighty one percent of all fractures in women aged 50 years and older can be attributed to osteoporosis [4]. Osteoporotic fractures, such as those of the hip, spine and wrist, often appear in older people following minor trauma. Hip fractures lead to rehabilitation problems and greatly decrease the quality of life [5,6].

Very little information is available on bone mineral density (BMD), fracture risk and their determinants among Canada’s First Nations people. The only existing publications focus on Manitoba’s Aboriginal population [7-12]. Retrospective studies using administrative health data have reported that members of First Nations in Manitoba had significantly higher osteoporotic fracture rates than non-Aboriginals [8,9]. Factors such as low socioeconomic status, diabetes [8] or the number of comorbidities and alcohol and drug use [9] were associated with higher fracture rates in these populations. Low calcium and vitamin D intake among Aboriginals, particularly older women [13], may also play a role.

Moreover, Aboriginal populations living in the northern regions of the globe are exposed to persistent organic pollutants through their traditional diet [14]. These include dioxin-like compounds (DLCs), similar in structure to 2,3,7,8-tetrachlorodibenzo-*p*-dioxin (TCDD), which are lipophilic, resistant to biodegradation and therefore bioaccumulate and bioamplify in aquatic food chains, where Aboriginals obtain part of their diet [15]. Yet the findings of *in vitro* studies [16] and experimental studies on rats [17-19] show that TCDD, the most toxic compound in the DLC family, has multiple effects on bone tissue, resulting in increased bone fragility. Few human studies have focused on the relationship between exposure to DLCs and bone quality/strength. Furthermore, while populations are exposed to a complex mixture of DLCs, only the plasma concentrations of certain dioxin-like PCBs (DL-PCBs) have been measured in participants in order to study the relationship between their concentrations and BMD or quantitative ultrasound (QUS) parameters [20-22].

The objective of this study was to investigate the relationship between plasma concentrations of DLCs or certain dioxin-like mono-ortho-substituted PCBs (DL-PCBs 105, 118 and 156) and calcaneal QUS parameters in Cree women of Eastern James Bay (Canada). Ultrasound bone measurement is a method used to assess bone strength that provides information that is complementary to BMD [23]. Recent studies demonstrate that QUS at the calcaneus can predict fractures as effectively as DXA in postmenopausal women and men over the age of 65 [24]. Values of QUS parameters are generally lower in osteoporotic patients than in healthy subjects [25]. This technique has several advantages: it is simple, fast, non-invasive, radiation-free and inexpensive. Furthermore, ultrasound bone measurement devices are portable and easy to use in remote, isolated regions, such as those inhabited by northern Aboriginal communities, where measurement of BMD cannot be performed by dual-energy x-ray absorptiometry (DXA).

## Methods

### Population

An environmental and health survey, entitled “Nituuchischaayihtitaau Aschii. Multi-community Environment- and Health Longitudinal Study in Eeyou Istchee”, was conducted from summer 2005 to summer 2009 in seven Cree communities located north of the 49^th^ parallel north, in the Eastern James Bay region of the province of Quebec (Canada). The objective of this study was to investigate the health of these Aboriginal populations and the risk factors affecting them [26,27].

Participants, stratified by age (0–7 years; 8–14 years; 15–39 years; ≥40 years) and gender, were selected using simple random sampling, without replacement, from the Beneficiary List in each community to create lists of potential participants to be contacted by recruiters. A weighting was attributed to each stratum to ensure that inferences could be drawn about the population. An initial list of subjects to be contacted was drawn up and all these people were contacted and asked to participate in the study. If they refused, a second list of participants was randomly created and recruitment continued in order to ensure the required number of subjects in each age group [28]. Owing to the low response rate (53.5%) in the first community visited, a correction to the predefined samples had to be taken into account in order to maintain the desired statistical power; the sample size in subsequent communities was therefore increased [29]. The total response rate of females (all age categories combined) varied between 34.4% and 70.9% depending on the community (Centre de Recherche du CHU de Québec, unpublished data). With respect to the non-participants, some could not be reached; some were undecided and withdrew, while others refused to participate. Others were excluded (unknown in the community, non-Cree, pregnant, disabled) [28,29].

All participants (aged 8 years and over) completed a series of questionnaires in *Eeyou Ayimuwin* or English and administered by the research staff or interviewers (selected in local communities and provided with appropriate training) [30,31]. They then underwent a clinical examination with biological samples and anthropometric measurements collected by research nurses.

All women aged 35 to 74 years who participated in the clinical examination were eligible for the calcaneal ultrasound measurement. A total of 254 women had QUS parameters measured. The volume of plasma collected from 249 women was sufficient to allow testing for DLCs and DL-PCBs.

### Bone measurements

Two QUS parameters were measured at the right calcaneus of Cree women using a portable Achilles Insight system (GE Healthcare Lunar, Madison, WI, USA): 1) speed of sound (SOS, m/s) and 2) broadband ultrasound attenuation (BUA, dB/MHz) [32]. The Stiffness Index (SI, %) was automatically calculated by the system from the two parameters using the manufacturer’s formula [SI% = (0.67*BUA) + (0.28*SOS) – 420]. A research nurse inspected and calibrated the system’s membranes daily using the phantom provided by the manufacturer. *In vitro* accuracy was assessed by taking a number of repeated measurements with the manufacturer’s phantom: the mean coefficients of variation (CVs) recorded for the SOS parameter ranged from 0.05 to 0.12% depending on the community.

### Anthropometric measurements

Body composition was measured using a bioelectric impedance analyzer (Tanita Corporation of America, IL, USA). Weight, lean body mass and body fat (kg), height, sitting height, waist and hip circumference (cm) were measured by research nurses using standardized techniques.

### Laboratory analyses

#### DLCs and DL-PCBs

Plasma samples were tested for DLCs and DL-PCBs at INSPQ’s Toxicology Laboratory (Quebec City, Canada). The luciferase reporter gene cell assay described by Medehouenou et al. [33] was used to measure AhR-mediated transcriptional activity elicited by plasma extracts and provide an integrated measure of DLCs. The limit of detection (LOD) was 30 pg TCDD-equivalents (EQ)/L. For quality control purposes, we used human plasma spiked with TCDD to obtain a concentration of 322 pg/L. The mean concentration of this sample tested 22 times was 361 pg TCDD-EQ/L (bias=12.1%) and the CV was 14%.

DL-PCBs (DL-PCB 105: 2,3,3’,4,4’-pentachlorobiphenyl; DL-PCB 118: 2,3’4,4’,5-pentachlorobiphenyl and DL-PCB 156: 2,3,3’,4,4’,5-hexachlorobiphenyl) were measured by gas chromatography–mass spectrometry (GC-MS) as described extensively in the project reports [34,35]. For samples from the first Cree community investigated, a liquid-liquid extraction was used followed by separation and quantification of PCB congeners by GC-MS using an Agilent 6890 Network GC system (Wilmington, DE, USA), fitted with an Agilent 7683 series automatic liquid sampler and an Agilent 5973 Network mass spectrometer. The recovery rate for the three DL-PCB congeners was over 90%. Coefficients of variation of 5.9%, 4.2% and 11.5% were obtained for DL-PCBs 105, 118 and 156 congeners respectively following repeated measures of standard reference material (SRM) 1589 (National Institute of Standards and Technology, Gaithersburg, MD, USA). The limit of detection for the three DL-PCBs was 0.02 μg/L [34]. For samples from the six other Cree communities investigated, a solid-phase extraction was used followed by GC-MS analysis with the same instrumentation as described above. Coefficients of variation of 7.4%, 7.7% and 9.7% were obtained for the three DL-PCB 105, 118 and 156 congeners respectively, following repeated measures of SRM 1589; the detection limit was 0.01 μg/L [35].

#### Lipids

Concentrations of total lipids in plasma samples were calculated using Phillips et al.’s formula [36]: [Total lipids (g/L) = (0.878*cholesterol mmol/L) + (0.885*triglycerides mmol/L) + 0.623]. Total cholesterol and triglyceride concentrations were determined by standard enzymatic methods. For the total cholesterol analysis, CVs of 1.5% and 1.3% were obtained for reference samples containing 2.87 and 6.67 mmol/L respectively. For the triglycerides analysis, CVs of 2.0% and 1.6% were noted for reference samples with concentrations of 1.07 and 2.32 mmol/L respectively [35].

Levels of omega-3 and omega-6 PUFAs were measured in erythrocyte membranes by liquid gas chromatography with flame ionisation detection at the Centre de Recherche sur les Maladies Lipidiques, CHU de Québec as previously described [34]. Omega-3 PUFAs refer to the following fatty acids: α-linolenic acid (C18:3n-3) + docosapentaenoic acid (C22:5n-3) + docosahexaenoic acid (C22:6n-3) + eicosapentaenoic acid (C20:5n-3) + (C18:4n-3) + (C20:3n-3) + (C20:4n-3). Omega-6 PUFAs refer to the following fatty acids: linoleic acid (C18:2n-6) + arachidonic acid (C20:4n-6) + (C18:3n-6) + (C20:2n-6) + (C20:3n-6) + (C22:2n-6) + (C22:4n-6) + (C22:5n-6).

Apolipoprotein B (Apo B) was measured by nephelometry using a BN ProSpec station (Dade Behring, Mississauga, ON). Control levels of 0.46 and 1.44 g/L for Apo B showed interassay CVs of 3.2% and 1.8% [34].

#### Metals/metalloids

Blood mercury levels (nmol/L) were measured by cold vapour atomic absorption spectrometry (Pharmacia) [34]. Cadmium (nmol/L), selenium (μmol/L) and lead (μmol/L) were measured by inductively coupled plasma mass spectrometry (ICP-MS). The blood samples were diluted in ammonium hydroxide and the metals converted to their elemental form by aspirating the sample into argon plasma before being identified and quantified by mass spectrometry. The samples were analysed using a Perkin Elmer Sciex Elan 6000 ICP-MS instrument). The LODs were 0.04 nmol/L for cadmium, 0.001 μmol/L for lead, 0.49 nmol/L for mercury and 0.09 μmol/L for selenium [35].

#### Other analyses

Glucose was measured by a hexokinase enzymatic assay employing the Roche Modular system. Interassay CVs were 1.6% and 1.4% for glucose at control values of 4.74 and 15.66 mmol/L respectively [35]. Alpha-tocopherol (vitamin E) was determined using a Waters high-pressure liquid chromatography system (Lachine, QC) equipped with an autosampler, a reverse phase column (Nucleosil ODS1) and a UV detector [35]. Serum levels of 25-hydroxyvitamin D (25OHD) were measured in the Biochemistry Laboratory at Montreal’s Hôpital St-Luc (CHUM) by a procedure that includes extraction and quantification by competitive radioimmunoassay using the IDS radioimmunoassay kit (Medicorp Inc., Montreal, QC) [35].

### Questionnaires

Questionnaires were used to collect information about participants’ socio-demographic characteristics (date and place of birth; level of education: none or primary school/secondary and higher), their lifestyle habits (tobacco use: yes/no; use of calcium and vitamin D supplements in the last 12 months: yes/no; physical activity: active/inactive; alcohol intake in the last 12 months: yes/no; milk consumption in the last month: yes/no), their gynaecological history (menopausal status: menopausal/non-menopausal; parity: yes/no; number of children; use of hormonal contraceptives: yes/no; use of hormone replacement therapy, HRT: yes/no) and their personal fracture history (yes/no). Women were considered menopausal if they had had no menstrual periods for one year before recruitment. Sodium intake (≤ 2300 mg vs > 2300 mg/day) was compiled based on answers to the 24-hour dietary recall and data on the sodium content of foods [37].

We consulted the medical records of participants to document causes of secondary osteoporosis (CSO: yes/no), namely the use of certain medications in the last year (corticosteroids, heparin, Dilantin, Prednisone, chemotherapy) or the diagnosis of diseases (Cushing’s disease, rheumatoid arthritis, bone cancer, gastrectomy, kidney failure, liver failure, thyroid and parathyroid diseases) that may contribute to bone loss [38].

Physical activity (PA) was assessed using the short form of the International Physical Activity Questionnaire [39]. A dichotomous variable was created based on the median value of the Total MET (metabolic equivalent)-minutes/week, calculated using the algorithm in the IPAQ document [39]. Values at or below the median (3954 MET-minutes/week) define an inadequate level of PA, or subjects considered inactive, while values above the median define an adequate level of PA, or subjects considered active.

### Statistical analyses

Our database comprised 249 participants. DLC concentrations were below the LOD (30 pg TCDD-EQ/L) for 16 participants. In these cases, we imputed a value between 0 and the LOD selected by simple random sampling with replacement. Of the 44 participants from the first community visited, four had DL-PCB 105 concentrations below the LOD, while one participant had a concentration of DL-PCB 156 below the LOD (LOD = 0.02 μg/L). Plasma concentrations of DL-PCBs 105, 118 and 156 below the LOD (0.01 μg/L) were obtained in 45, 7 and 12 participants respectively among the 205 residents of the other six communities. In these cases, we imputed a value equal to LOD/2, that is, 0.01 μg/L for participants from the first community and 0.005 μg/L for participants from the other communities.

Descriptive statistics (mean, standard error, minimum and maximum for quantitative variables; sample size and % per modality for qualitative variables) of the variables were generated for all participants.

The association between DLCs or each DL-PCB and QUS parameters was examined by simple and multiple linear regressions. Box-Cox procedures were used to resolve problems encountered with the hypotheses of normality and/or homoscedasticity in the multiple regression models for the dependent variables SOS and SI, which were subsequently log-transformed. Pearson’s correlation coefficients were calculated between the QUS parameters and the main exposure variables. The confounding effect of different factors on the relation between DLCs or DL-PCBs and QUS parameters was investigated. Secondary independent variables were tested to determine whether or not they should be included in the initial multiple regression models. Only those with a p value ≤ 0.20 (in simple linear regression analysis) were included. Multicollinearity between the variables of the initial regression models was tested to ensure that variables were not redundant. To avoid multicollinearity caused by the introduction of highly intercorrelated variables in the same linear regression model, we proposed for each dependent variable (SOS, BUA and SI) separate models that comprised only one of the main exposure variables (either DLCs, DL-PCB 105, DL-PCB 118 or DL-PCB 156). All potentially confounding variables were entered in the multiple regression model and those that did not change the value of β coefficient of the main exposure variable by more than 10% were removed one by one, starting with the variable that accounted for the least variation in the dependent variable. In the case of highly intercorrelated anthropometric variables, when one of these variables was not identified as a confounding factor, and therefore not retained in the final model, the initial model was rebuilt with the other variable.The final adjustment of the models was for the total plasma lipid concentration [40]. We also constructed a second set of multivariate models (models II in Additional files 1, 2 and 3) adjusted for the same confounders and covariates. A p value of < 0.05 in a bilateral situation is considered statistically significant. The software used was SAS version 9.2 (SAS Institute Inc., Cary, NC, USA).

The project was approved by the Research Ethics Committees of Université Laval and McGill University, in partnership with McMaster University, and by the Research Committee of the Cree Board of Health and Social Services of James Bay. Participation in the study was voluntary and a consent form was signed by each participant. All information concerning the participants was kept strictly confidential.

## Results

With the weighting applied, the sample of 249 women (102 menopausal women and 147 non-menopausal women) could be extrapolated to a population of 2056 women (883 menopausal women and 1173 non-menopausal women).

QUS parameters, plasma DLC and DL-PCB concentrations as well as selected characteristics of the Cree women are shown in Table 1 and Additional file 4. Most participants were non-menopausal, had a secondary level education or higher, had had children, were sedentary, non-smokers and did not drink alcohol. A small proportion of women had used hormonal contraception or HRT, had taken supplements (calcium and vitamin D) in the 12 months prior to the study and had a history of fracture and conditions and/or medications identified as CSO.

**Table 1:** Characteristics of participants

**Characteristic**	**N**	**AM**^**(a) **^**± SD**^**(b)**^	**Range**^**(c)**^	**GM**^**(d) **^**(95%-CI)**^**(e)**^
**Dependant variables**				
SOS (m/s)	249	1545.10 ± 39.21	1466.03-1665.22	
BUA (dB/MHz)	249	118.05 ± 16.81	67.67-169.35	
SI (%)	249	91.24 ± 20.59	46-151	
**Main exposition variables**				
DLC (pg TCDD-EQ/L)	249	234.13 ± 319.05	0.95-3115	124.31 (106.94-144.50)
DL-PCB 105 (μg/L)	249	0.09 ± 0.15	0.01-1.28	0.035 (0.03-0.04)
DL-PCB 118 (μg/L)	249	0.52 ± 0.90	0.01-8.35	0.19 (0.16-0.23)
DL-PCB 156 (μg/L)^(f)^	223	0.29 ± 0.42	0.01-3.15	0.12 (0.10-0.14)
**Other variables**				
Age (years)	249	47.98 ± 10.72	35-74	
Weight (kg)	248	91.46 ± 18.72	46.3-171.6	
Lean mass (kg)	248	48.63 ± 6.48	25.8-75.2	
Height (cm)	249	160.27 ± 6.03	134-175	
Height in seated position (cm)	245	85.73 ± 3.24	74.7-93.85	
Number of children	246	3.72 ± 2.77	0-13	
Omega-3 PUFAs (%)	248	6.87 ± 1.38	2.16-13.9	
Omega-6 PUFAs (%)	248	30.24 ± 1.73	22.44-33.76	
Vitamin D (nmol/L)	249	59.03 ± 19.77	23-138	
Glucose (mmol/L)	249	7.17 ± 8.98	3.0-21.8	
Apolipoprotein B (g/L)	249	0.97 ± 1.17	0.31-2.62	
Total lipid (g/L)	249	6.19 ± 1.28	3.49-14.64	
Mercury (nmol/L)	249	51.09 ± 61.38	0.60-393.84	26.49 (22.67-30.94)
Selenium (μmol/L)	249	2.21 ± 0.42	1.3- 4.9	2.18 (2.14-2.23)
Lead (μmol/L)	249	0.29 ± 0.32	0.02-2.5	0.18 (0.16-0.21)
	**N**	**N weighted (%)**		
Menopausal status	249	2056.13		
Menopausal	102	882.589 (42.92)		
Non-menopausal	147	1173.54 (57.08)		
Level of education	248	2044.31		
None or primary school	74	620.02 (30.33)		
Secondary or higher	174	1424.29 (69.67)		
Supplements use^(g)^	232	1881.49		
Yes	39	272.365 (14.48)		
No	193	1609.13 (85.52)		
Physical activity	243	2021.47		
Active	118	986.11 (48.78)		
Inactive	125	1035.37 (51.22)		
Tobacco use	248	2042.24		
Yes	94	726.82 (35.59)		
No	154	1315.43 (64.41)		
Sodium intake	249	2056.13		
≤ 2300 mg	108	876.94 (42.65)		
> 2300 mg	141	1179.19 (57.35)		

^a^ Unweighted arithmetic mean.

^b^ Standard deviation;

^c^ Minimum-maximum;

^d^ Unweighted geometric mean;

^e^ 95%-Confidence interval of geometric mean;

^f^ Variable not measured in one community;

^g^ Calcium and vitamin D supplements.

Most of the Cree women (96.9%) had a waist circumference ≥ 88 cm (abdominal obesity according to Health Canada) [41]; the median value was 112 cm. With respect to body mass index (BMI, kg/m^2^), according to Health Canada criteria [41], only 3.5% of Cree women had a normal BMI (between 18.5 and 24.9), 19.2% were overweight (BMI between 25.0 and 29.9 kg/m^2^) and 77.3% were obese (BMI ≥ 30 kg/m^2^). Among Cree women, 9.0% had a height of less than 153 cm, which has been associated with osteoporosis [42].

The maximum blood lead level of 0.32 μmol/L noted in women from the Quebec City area was exceeded by 26.9% of Cree women [43], while 14.2% exceeded Canada’s alert level of 0.48 μmol/L [44].

With regard to blood mercury levels, 64.5% of Cree women exceeded the maximum blood mercury level of 16 nmol/L observed in women from the Quebec City area [43], whereas 13.2% exceeded Canada’s alert level of 99.7 nmol/L [45].

Only 1.9% of participants exhibited a blood selenium concentration exceeding the maximum level (3.6 μmol/L) recorded in women from the Quebec City area [43] and 1.8% showed a blood cadmium concentration over the maximum value in the Quebec City inhabitants (55 nmol/L) [43].

With respect to serum levels of vitamin D (25OHD), 2.3% of Cree women had a level considered as critical (< 27.5 nmol/L) [46]; 14.6% displayed inadequate levels (< 37.5 nmol/L), while 39.5% showed a level considered minimal (< 50 nmol/L) [38].

Fasting glucose levels were considered normal (< 6.1 mmol/L) in 56.0% of Cree women, while 44.0% had levels corresponding to hyperglycemia (≥ 6.1 mmol/L). For 26.7% of Cree women, glucose levels could correspond to type 2 diabetes (≥ 7.0 mmol/L) according to the Canadian Diabetes Association [47].

Pearson’s r correlation coefficients revealed that QUS parameters were negatively and significantly correlated with DLCs, DL-PCBs and age, while the three DL-PCBs were positively and significantly correlated with one another and with age (see Additional file 5).

The final multivariate models of each QUS parameter, constructed for each of the main exposure variables (DLCs or DL-PCBs), are presented in Tables 2, 3 and 4. DL-PCBs 105 and 118 were significantly and negatively associated with SI (log); these associations persisted even after adjustment for several confounding factors. Hence the SI (log) decreased by −0.22% (p=0.0414) and −0.04% (p=0.0483) for every one-unit increase (1 μg/L) in plasma concentrations of DL-PCBs 105 and 118, respectively (Table 4). In QUS parameters models built for each of the main exposure variables and adjusted for the same factors (see Additional files 1, 2 and 3, models II), DL-PCB 105 was negatively and significantly associated with BUA (Additional file 2), whereas DL-PCBs 105 and 118 were negatively and significantly associated with SI (log) (Additional file 3).

**Table 2:** Results of explanatory multivariate analysis: SOS (log) models (I)

**SOS (log) model**	**Main exposure variable**							
	**DLCs (pg TCDD-EQ/L)**		**DL-PCB 105 (μg/L)**		**DL-PCB 118 (μg/L)**		**DL-PCB 156 (μg/L)**	
**N**	240		220		241		202	
**R**^**2 **^**(Adjusted R**^**2**^**)**	0.3086 (0.2721)		0.3470 (0.2990)		0.3070 (0.2737)		0.3123 (0.2725)	
	**Regression coefficient (SE)**^**(a)**^	**p-value**	**Regression coefficient (SE)**^**(a)**^	**p-value**	**Regression coefficient (SE)**^**(a)**^	**p-value**	**Regression coefficient (SE)**^**(a)**^	**p-value**
**Variables**	**DLCs**	0.4012	**DL-PCB 105**	0.2008	**DL-PCB 118**	0.1440	**DL-PCB 156**	0.2534
	0.009 (0.01)		−18.66 (14.54)		−3.40 (2.32)		−6.38 (5.57)	
Age (years)	−1.02 (0.30)	0.0007	−1.03 (0.29)	0.0005	−1.26 (0.19)	<0.0001	−1.44 (0.22)	<0.0001
Weight (kg)	0.007 (0.09)	0.9409	NR		NR		NR	
Height (cm)	−0.49 (0.28)	0.0852	−0.84 (0.28)	0.0027	−0.54 (0.27)	0.0466	−0.75 (0.29)	0.0118
Number of children	NR		0.09 (0.63)	0.8854	NR		−0.10 (0.61)	0.8749
Omega-3 PUFAs (%)	1.27 (1.27)	0.3176	2.19 (1.30)	0.0928	1.79 (1.28)	0.1620	2.11 (1.33)	0.1143
Vitamin D (nmol/L)	NR		0.07 (0.10)	0.4498	NR		NR	
Mercury (nmol/L)	0.02 (0.03)	0.6270	0.03 (0.04)	0.4081	0.03 (0.03)	0.3461	0.05 (0.04)	0.2162
Selenium (μmol/L)	2.13 (3.86)	0.5820	0.39 (0.53)	0.4626	4.30 (3.70)	0.2463	2.74 (3.79)	0.4696
Glucose (mmol/L)	NR		3.51 (3.64)	0.3354	0.46 (0.51)	0.3637	NR	
Apolipoprotein B (g/L)	−5.70 (3.90)	0.1454	NR		−5.81 (3.86)	0.1340	NR	
Menopausal status	−8.62 (4.89)	0.0791	−10.34 (4.92)	0.0368	NR		NR	
Level of education	9.41 (3.86)	0.0156	10.15 (3.91)	0.0102	7.62 (3.84)	0.0486	8.06 (4.13)	0.0526
Smoking status	NR		−6.75 (3.39)	0.0480	NR		−6.44 (3.59)	0.0741
Physical activity	5.49 (2.92)	0.0610	6.10 (3.03)	0.0454	5.97 (2.91)	0.0416	NR	
Supplements use ^(b)^	NR		−5.50 (4.82)	0.2550	NR		−0.54 (5.19)	0.9177
Total lipid^(c)^ (g/L)	0.51 (1.17)	0.6661	0.94 (1.27)	0.4582	0.47 (1.19)	0.6927	1.52 (1.33)	0.2532

^a^ Regression coefficients and standard errors values are multiplied by 10^3^;

^b^ Calcium and vitamin D supplements;

^c^ Final adjustment for the total plasma lipid concentration;

NR – Variable not retained in the model (non-confounding factor; proportional change of the regression coefficient of the main exposure variable < 10%).

**Table 3:** Results of explanatory multivariate analysis: BUA models (I)

**BUA model**	**Main exposure variable**							
	**DLCs (pg TCDD-EQ/L)**		**DL-PCB 105 (μg/L)**		**DL-PCB 118 (μg/L)**		**DL-PCB 156 (μg/L)**	
**N**	226		239		244		203	
**R**^**2 **^**(Adjusted R**^**2**^**)**	0.3680 (0.3324)		0.3579 (0.3356)		0.3496 (0.3303)		0.3424 (0.3188)	
	**Regression coefficient (SE)**	**p-value**	**Regression coefficient (SE)**	**p-value**	**Regression coefficient (SE)**	**p-value**	**Regression coefficient (SE)**	**p-value**
**Variables**	**DLCs**	0.4275	**DL-PCB 105**	0.1060	**DL-PCB 118**	0.0841	**DL-PCB 156**	0.2649
	0.005 (0.007)		−13.23 (8.15)		−2.42 (1.40)		−3.79 (3.39)	
Age (years)	−0.73 (0.19)	0.0002	−0.78 (0.13)	<0.0001	−0.83 (0.12)	<0.0001	−0.74 (0.13)	<0.0001
Weight (kg)	0.04 (0.06)	0.5404	NR		NR		NR	
Lean mass (kg)	NR		0.18 (0.17)	0.2924	NR		NR	
Height in seated position (cm)	0.19 (0.37)	0.6181	0.13 (0.36)	0.7163	NR		NR	
Omega-6 PUFAs (%)	−1.43 (0.66)	0.0317	−0.72 (0.63)	0.2521	−0.75 (0.62)	0.2268	NR	
Mercury (nmol/L)	NR		NR		0.01 (0.02)	0.4820	0.01 (0.02)	0.6159
Selenium (μmol/L)	−1.998 (2.41)	0.4075	NR		NR		NR	
Lead (μmol/L)	2.78 (3.66)	0.4487	NR		NR		NR	
Number of children	NR		0.42 (0.39)	0.2882	0.48 (0.38)	0.2144	0.43 (0.38)	0.2663
Menopausal status	−2.63 (3.20)	0.4134	NR		NR		NR	
Level of education	5.95 (2.43)	0.0150	4.63 (2.42)	0.0573	5.55 (2.37)	0.0202	5.28 (2.54)	0.0386
Supplements use^(a)^	−3.58 (2.82)	0.2063	NR		NR		−2.53 (3.25)	0.4369
Sodium intake	2.56 (2.02)	0.2062	NR		NR		NR	
Total lipid^(b)^ (g/L)	−0.93 (0.72)	0.1971	−0.02 (0.79)	0.9810	−0.24 (0.78)	0.7558	0.001 (0.83)	0.9991

^a^ Calcium and vitamin D supplements;

^b^ Final adjustment for the total plasma lipid concentration;

NR – Variable not retained in the model (non-confounding factor; proportional change of the regression coefficient of the main exposure variable < 10%).

**Table 4:** Results of explanatory multivariate analysis: SI (log) models (I)

**SI (log) model**	**Main exposure variable**							
	**DLCs (pg TCDD-EQ/L)**		**DL-PCB 105 (μg/L)**		**DL-PCB 118 (μg/L)**		**DL-PCB 156 (μg/L)**	
**N**	245		246		244		203	
**R**^**2 **^**(Adjusted R**^**2**^**)**	0.3935 (0.3675)		0.3631 (0.3471)		0.3932 (0.3725)		0.3766 (0.3475)	
	**Regression coefficient (SE)**^**(a)**^	**p-value**	**Regression coefficient (SE)**^**(a)**^	**p-value**	**Regression coefficient (SE)**^**(a)**^	**p-value**	**Regression coefficient (SE)**^**(a)**^	**p-value**
**Variables**	**DLCs**	0.3806	**DL-PCB 105**	0.0414	**DL-PCB 118**	0.0483	**DL-PCB 156**	0.1379
	0.07 (0.08)		−219.24 (106.90)		−37.84 (19.07)		−69.81 (46.87)	
Age (years)	−13.87 (2.58)	<0.0001	−12.74 (1.49)	<0.0001	−12.55 (1.57)	<0.0001	−12.21 (1.76)	<0.0001
Weight (kg)	0.11 (0.75)	0.8815	NR		NR		NR	
Height (cm)	−2.09 (2.36)	0.3762	−0.75 (2.18)	0.7321	−2.38 (2.23)	0.2860	−2.60 (2.50)	0.2994
Number of children	NR		6.22 (5.09)	0.2232	4.71 (5.00)	0.3477	25.2 (5.17)	0.6264
Omega-3 PUFAs (%)	18.36 (9.89)	0.0646	NR		23.90 (9.94)	0.0170	17.64 (11.36)	0.1221
Vitamin D (nmol/L)	0.57 (0.72)	0.4298	NR		NR		NR	
Mercury (nmol/L)	NR		0.22 (0.25)	0.3769	NR		0.31 (0.31)	0.3215
Glucose (mmol/L)	NR		NR		2.88 (4.18)	0.4914	NR	
Menopausal status	−26.05 (40.99)	0.5256	NR		NR		NR	
Level of education	95.19 (31.70)	0.0030	NR		71.25 (31.37)	0.0240	72.72 (34.94)	0.0388
Smoking status	−36.58 (27.40)	0.1831	NR		NR		NR	
Supplements use^(b)^	NR		NR		NR		−12.67 (43.91)	0.7733
Total lipid^(c)^ (g/L)	−5.28 (9.51)	0.5796	7.31 (10.41)	0.4829	3.04 (10.38)	0.7696	7.19 (11.28)	0.5244

^a^ Regression coefficients and standard errors values are multiplied by 10^3^;

^b^ Calcium and vitamin D supplements;

^c^ Final adjustment for the total plasma lipid concentration;

NR – Variable not retained in the model (non-confounding factor; proportional change of the regression coefficient of the main exposure variable < 10%).

Several variables were identified as confounders of associations between exposure variables and QUS parameters. Some confounding factors were significantly associated with SOS (log): age (in all models), level of education (DLC, DL-PCB 105 and DL-PCB 118 models), height (all DL-PCB models), PA (DL-PCB 105 and DL-PCB 118 models), smoking status and menopausal status (DL-PCB 105 model) (Table 2). In general, SOS (log) decreased with increasing age, height, menopausal status and smoking status and SOS (log) increased with increasing level of education and physical activity.

Age (in all models), level of education (DLC, DL-PCB 118 and DL-PCB 156 models) and % of omega-6 PUFAs (DLC model) were significantly associated with BUA (Table 3). In general, BUA decreased with increasing age and % of omega-6 PUFAs, and increased with increasing level of education.

Lastly, age (in all models), level of education (DLC, DL-PCB 118 and DL-PCB 156 models) and % of omega-3 PUFAs (DL-PCB 118 model) were significantly associated with SI (Table 4). In general, SI (log) decreased with increasing age, and increased with an increase in the total % of omega-3 PUFAs and with increasing level of education.

## Discussion

This is the first population study to investigate the relationship between total DLC concentration measured with an AhR responsive reporter gene assay and bone quality parameters [48]. In Cree women aged 35 to 74 years of Eastern James Bay, DL-PCBs 105 and 118 were negatively and significantly associated with SI. However, we did not find any association between total DLC concentrations measured by the reporter gene bioassay and QUS parameters, which suggests that the effect of PCBs is not linked to activation of the AhR signalling pathway. Alternatively, the lack of association could also be due to the lower precision of the bioassay measurement compared to that of GC-MS measurements.

Three studies have been conducted on the relation between DL-PCBs and QUS bone parameters, with quite controversial findings. In a group of Swedish men from the general population (n = 115, age 40–75 years), Glynn et al. [20] reported a positive association at the limit of significance (β = 0.0044; p = 0.05) between serum concentrations of DL-PCB 167 and whole-body BMD (measured by DXA), after adjustment for several confounding factors. However, no association was observed between DL-PCB 167 and BUA or SOS parameters measured by ultrasound at the left calcaneus [20]. No association was observed between DL-PCBs 105, 118, 156 and QUS parameters or lumbar spine, femoral neck or whole-body BMD [20].

Exploratory multivariate analyses conducted by Côté et al. [21] revealed that plasma concentrations of DL-PCB 156 were negatively associated with QUS parameters measured in 153 peri- and postmenopausal Inuit women from Nuuk in Greenland (SOS model: β = −22.68, p = 0.014; BUA model: β = −8.12, p = 0.028; SI model: β = −11.95, p = 0.009) [21]. However, DL-PCBs 105 and 118 were not associated with QUS parameters [21].

Hodgson et al. [22] found a negative association between serum DL-PCB 118 concentration and forearm BMD measured using DXA (β = −0.00024, p = 0.002) in the dominant arm of 154 men (60–81 year old) from the Swedish OSCAR cohort study. The odds ratio for low BMD of 1.06 per 10 pg/mL DL-PCB 118 was significant (95% CI: 1.01-1.12) [22]. However, in women (n = 167), DL-PCB 118 was positively associated with BMD (β = 0.00008, p = 0.045). In this study, no association was observed between DL-PCBs 105, 156, 157, 167 and BMD; the authors considered the statistical power to be limited and a large proportion of variance was not explained by regression models [22].

In additional analyses, we performed logistic regression using medians for SI (91%), DL-PCB 105 (0.03 μg/L) and DL-PCB 118 (0.18 μg/L) as cut-off values between “low” and “high” categories. Statistically-significant odds ratios (OR) of 1.69 for low SI at high DL-PCB 105 exposure (95% CI: 1.34-2.13) and 1.36 for low SI at high DL-PCB 118 exposure (95% CI: 1.06-1.73) were obtained in models adjusted for all variables indicated in Table 4, confirming the associations obtained by multiple linear regression. However, the choice of medians as a cut-off values to define “low” and “high” categories of SI and the main exposure variables is arbitrary and does not convey the same meaning in different populations. Cut-off values for QUS parameters based on pathophysiological considerations are not available in the literature.

The geometric means (GMs) of plasma DL-PCB concentrations among Cree women were higher than those reported in the 2007–2009 Canadian Health Measures Survey for women aged 40 to 79 years and representative of the general Canadian population [49]. However, mean plasma concentrations of DLCs in Cree women estimated by the reporter gene assay were lower than those of women of childbearing age (Inuit, Dene, Caucasian) in other regions in Northern Canada [14].

Explanatory multivariate analyses revealed that several other factors were significantly associated with QUS parameters. Negative associations were found with age, height, smoking status, menopausal status and omega-6 PUFAs, whereas positive associations were noted with level of education, PA and % of omega-3 PUFAs.

Omega-3 PUFAs measured as a % of total fatty acids in erythrocyte membrane phospholipids in Cree women were positively and significantly associated with SI, while the % of omega-6 PUFAs was negatively associated with the BUA parameter. As observed following additional analyses, the omega-3/omega-6 PUFA ratio was positively and significantly associated with BUA and SI (log). To our knowledge, associations between omega-3 and omega-6 PUFAs and calcaneal QUS parameters have not been reported previously. A high ratio of omega-6 to omega-3 PUFAs has been associated with low BMD [50], while a higher dietary intake of omega-3 PUFAs had a protective effect against bone loss [51,52].

Our study has several strengths. Firstly, the representativeness of our population sample was assured by the recruitment strategy and the weighting scheme of the study, which took non-response and refusal to participate rates into account. Hence the findings can be generalized to the entire female Cree population aged 35 to 74 years living in Eastern James Bay communities. Secondly, we took into consideration a large number of potential confounding factors of the association between the main exposure variables (DLCs, DL-PCBs) and QUS parameters. Several of these factors were investigated for the first time (% omega-3 and omega-6 PUFAs, mercury, selenium, lead, glucose, apolipoprotein B). Thirdly, measurement biases in the dependent variables or exposure variables are relatively unlikely. Data collection in the field (ultrasound measurements, anthropometric measurements, biological samples) was performed by research nurses using standardized techniques. Completed questionnaires were reviewed by members of the research team to ensure that all the questions had been completely and properly answered [30,31]. Individual levels of exposure to contaminants were measured using standardized methods in the same laboratory (INSPQ) which has a quality control system accredited according to ISO/CEI 17025, CAN-P-43 and ILAC G-13 standards. All laboratory analyses (plasma or blood) were carried out, with two exceptions (PUFAs and 25OHD), at the INSPQ laboratory. Measurement accuracy complied with applicable standards.

Our study has also certain limitations. Firstly, the main methodological limitation is its cross-sectional design, with exposure and the dependent variable measured at the same time (a single measurement for each subject), such that the temporal sequence of cause and effect cannot be determined. However, due to the fact that DLCs and DL-PCBs accumulate in the body with age, plasma concentrations in Cree women reflect their lifelong exposure. Secondly, the participation rate in the study varied largely between communities, which may suggest a selection bias. However, this bias is quantitatively unimportant, since it is unlikely that the characteristics of the subjects included in the study are different from those of all eligible persons. The lists of beneficiaries which were used to build the list of contact persons were completed as necessary with information from other local listings; moreover, the investigated communities are small and live in reserves, which facilitated the identification and location of their members. Regarding the non-response and refusal to participate in the study, it is unlikely that these subjects have different levels of plasma DL-PCBs congeners or QUS parameter values, compared with participants; the recruitment of participants was made for a survey of general health and not specifically for the purpose of our study. Thirdly, the number of participants with QUS measurements was relatively small, limiting our capacity to observe an association between DLC concentrations determined by the reported gene assay and QUS parameters, especially considering that this measure is relatively imprecise compared to analytical chemistry based data. Fourthly, while we took a maximum number of potential confounding factors into account given the size of our sample, a residual confounding effect cannot be ruled out. The determinants of bone strength are multigenic and multifactorial and other factors (genetic, nutritional, environmental) not measured in our study could have decreased the residual variance in multiple regression models. Lastly, two variables that are known to influence bone quality could not be thoroughly assessed. The assessment of dietary calcium intake considered only milk consumption. Physical activity was evaluated using the short form of the IPAQ [39], an instrument that proved difficult to administer in Cree communities, even though its feasibility and validity has been demonstrated in general populations (adults aged 15 to 69 years) of 12 countries [53]. Participants in our study had trouble quantifying their activities, especially the duration and intensity of activities during the week prior to the study [54]. We believe that the proportion of active Cree women was probably overestimated.

## Conclusions

We observed negative associations between SI values and plasma concentrations of two dioxin-like PCB congeners in women from this Aboriginal population. Our study does not allow causal inference and residual confounding may still be present. Therefore, we do not believe that dietary recommendations to reduce PCB exposure are warranted at this time. In multivariate models, associations were also noted with dietary and lifestyle factors, suggesting avenues to improve bone quality in this population, such as smoking cessation and increases in omega-3 PUFA intake and physical activity.

## Abbreviations

AhR: aryl hydrocarbon receptor; AM: arithmetic mean; Apo B: Apolipoprotein B; BMD: bone mineral density; BMI: body mass index; DL-PCB: dioxin-like polychlorinated biphenyl; BUA: broadband ultrasound attenuation; CHUM: Centre Hospitalier de l’Université de Montréal; CHU de Québec: Centre Hospitalier Universitaire de Québec; 95% CI: 95% confidence interval; CSO: causes of secondary osteoporosis; CV: coefficient of variation; dB/MHz: decibels per microhertz; DLCs: dioxin-like compounds; DL-PCBs: dioxin-like polychlorinated biphenyls; DXA: dual-energy x-ray absorptiometry; GC-MS: gas chromatography–mass spectrometry; GM: geometric mean; g/L: grams per litre; HRT: hormone replacement therapy; ICP-MS: inductively coupled plasma mass spectrometry; INSPQ: Institut National de Santé Publique de Québec; MET: metabolic equivalent; m/s: metres per second; μg/L: micrograms per litre; μmol/L: micromoles per litre; mmol/L: millimoles per litre; nmol/L: nanomoles per litre; NR: not retained; LOD: limit of detection; OR: odds ratio; PUFA: polyunsaturated fatty acid; PA: physical activity; %: percentage; pg TCDD-EQ/L: picograms TCDD-equivalents per litre; QC: quality control; QUS: Quantitative UltraSonography; RR: relative risk; SOS: speed of sound; SRM: standard reference material; SI: Stiffness Index; TCDD: 2,3,7,8-tetrachlorodibenzo-*p*-dioxin; UV: ultra violet; 25OHD: 25-hydroxyvitamin D.

## Competing interests

The authors declare that they have no competing interests.

## Authors’ contributions

ED, PA, SD and EN conceived the study design. ACP participated to data collection, performed the statistical analysis, interpreted the data, and drafted the manuscript. PA interpreted the data and helped draft the manuscript. All authors revised the manuscript critically. All authors approved the final version.

## Supplementary Material

Additional file 1 Multivariate analysis: SOS (log) models (II). Click here for file

Additional file 2 Multivariate analysis: BUA models (II). Click here for file

Additional file 3 Multivariate analysis: SI (log) models (II). Click here for file

Additional file 4 Other characteristics of participants. Click here for file

Additional file 5 Pearson’s correlation coefficients between age, plasma concentrations of DLC and DL-PCBs, and QUS parameters in Cree women. Click here for file
