# Supplementary material for: Dioxin-like compounds and bone quality in Cree women of Eastern James Bay (Canada): a cross-sectional study
Source: Environ Health. 2013 Jul 2;12:54. doi: 10.1186/1476-069X-12-54 (PMC3704868; doi:10.1186/1476-069X-12-54)
Supplement: Additional file 1 — Multivariate analysis: SOS (log) models (II). [file 1476-069X-12-54-S1.docx]

**Additional file 1**

Multivariate analysis: SOS (log) models (II).

| **SOS (log) model** | **Main exposure variable** | | | | | | | |
| --- | --- | --- | --- | --- | --- | --- | --- | --- |
|  | **DLC** (pg TCDD-EQ/L) | | **DL-PCB 105** (μg/L) | | **DL-PCB 118** (μg/L) | | **DL-PCB 156** (μg/L) | |
| **N**  **R^2^ (Adjusted R^2^)** | 242  0.3093 (0.2699) |  | 242  0.3122 (0.2730) |  | 242  0.3125 (0.2733) |  | 217  0.3167 (0.2730) |  |
|  |  |  |  |  |  |  |  |  |
|  | **Regression coefficient (SE)^(a)^** | **p-value** | **Regression coefficient (SE)^(a)^** | **p-value** | **Regression coefficient (SE)^(a)^** | **p-value** | **Regression coefficient (SE)^(a)^** | **p-value** |
|  |  |  |  |  |  |  |  |  |
| **Variables** | **DLC**  0.006 (0.01) | 0.5602 | **DL-PCB 105**  -15.48 (13.43) | 0.2502 | **DL-PCB 118**  -2.71 (2.28) | 0.2359 | **DL-PCB 156**  -5.78 (5.39) | 0.2845 |
|  |  |  |  |  |  |  |  |  |
| Age (years) | -1.27 (0.31) | <0.0001 | -1.15 (0.29) | <0.0001 | -1.15 (0.29) | <0.0001 | -1.14 (0.30) | 0.0002 |
| Weight (kg) | -0.03 (0.09) | 0.7036 | -0.02 (0.09) | 0.8465 | -0.02 (0.09) | 0.8020 | -0.01 (0.097) | 0.9492 |
| Height (cm) | -0.52 (0.280 | 0.0638 | -0.59 (0.28) | 0.0348 | -0.58 (0.28) | 0.0370 | -0.54 (0.297) | 0.0709 |
| Number of children | -0.14 (0.60) | 0.8193 | 0.21 (0.61) | 0.7320 | 0.19 (0.60) | 0.7528 | 0.14 (0.60) | 0.8222 |
| Omega-3/omega-6 PUFAs | 39.99 (31.85) | 0.2106 | 47.48 (31.84) | 0.1373 | 48.34 (31.92) | 0.1313 | 46.55 (32.95) | 0.1592 |
| Vitamin D (nmol/L) | 0.04 (0.09) | 0.6208 | 0.04 (0.09) | 0.6851 | 0.04 (0.09) | 0.6568 | 0.11 (0.10) | 0.2658 |
| Mercury (nmol/L) | 0.01 (0.03) | 0.6987 | 0.02 (0.03) | 0.5249 | 0.02 (0.03) | 0.5153 | 0.03 (0.04) | 0.4182 |
| Selenium (μmol/L) | 1.38 (3.79) | 0.7165 | 3.12 (3.59) | 0.3846 | 3.30 (3.62) | 0.3628 | 0.20 (3.74) | 0.5941 |
| Menopausal status | -7.16 (4.83) | 0.1398 | -8.03 (4.77) | 0.0933 | -7.92 (4.76) | 0.0973 | -8.85 (4.99) | 0.0775 |
| Level of education | 8.81 (3.77) | 0.0202 | 7.57 (3.75) | 0.0446 | 7.51 (3.75) | 0.0466 | 7.60 (4.01) | 0.0595 |
| Smoking status | -5.46 (3.27) | 0.0965 | -5.17 (3.26) | 0.1137 | -5.27 (3.26) | 0.1070 | -4.67 (3.47) | 0.1792 |
| Total lipid^(b)^ (g/L) | 1.14 (1.23) | 0.3547 | 1.41 (1.24) | 0.2551 | 1.37 (1.23) | 0.2695 | 1.44 (1.31) | 0.2750 |

^a^ Regression coefficients and standard errors values are multiplied *10^3^;

^b^ Final adjustment for the total plasma lipid concentration.
