# Supplementary material for: Dioxin-like compounds and bone quality in Cree women of Eastern James Bay (Canada): a cross-sectional study
Source: Environ Health. 2013 Jul 2;12:54. doi: 10.1186/1476-069X-12-54 (PMC3704868; doi:10.1186/1476-069X-12-54)
Supplement: Additional file 2 — Multivariate analysis: BUA models (II). [file 1476-069X-12-54-S2.docx]

**Additional file 2**

Multivariate analysis: BUA models (II).

| **BUA model** | **Main exposure variable** | | | | | | | |
| --- | --- | --- | --- | --- | --- | --- | --- | --- |
|  | **DLC** (pg TCDD-EQ/L) | | **DL-PCB 105** (μg/L) | | **DL-PCB 118** (μg/L) | | **DL-PCB 156** (μg/L) | |
| **N**  **R^2^ (Adjusted R^2^)** | 242  0.3542 (0.3174) |  | 242  0.3670 (0.3309) |  | 242  0.3640 (0.3278) |  | 217  0.3686 (0.3281) |  |
|  |  |  |  |  |  |  |  |  |
|  | **Regression coefficient (SE)** | **p-value** | **Regression coefficient (SE)** | **p-value** | **Regression coefficient (SE)** | **p-value** | **Regression coefficient (SE)** | **p-value** |
|  |  |  |  |  |  |  |  |  |
| **Variables** | **DLC**  0.004 (0.01) | 0.5542 | **DL-PCB 105**  -19.38 (8.71) | 0.0270 | **DL-PCB 118**  -2.92 (1.48) | 0.0501 | **DL-PCB 156**  -4.18 (3.50) | 0.2340 |
|  |  |  |  |  |  |  |  |  |
| Age (years) | -0.90 (0.20) | <0.0001 | -0.79 (0.19) | <0.0001 | -0.81 (0.19) | <0.0001 | -0.81 (0.19) | <0.0001 |
| Weight (kg) | 0.06 (0.058) | 0.3451 | 0.07 (0.06) | 0.2138 | 0.06 (0.06) | 0.2602 | 0.03 (0.06) | 0.6953 |
| Height (cm) | 0.05 (0.18) | 0.7787 | -0.02 (0.18) | 0.9262 | 0.00007 (0.18) | 0.9997 | 0.09 (0.19) | 0.6424 |
| Number of children | 0.18 (0.39) | 0.6538 | 0.54 (0.39) | 0.1671 | 0.48 (0.39) | 0.2160 | 0.40 (0.39) | 0.3111 |
| Omega-3/omega-6 PUFAs | 35.50 (20.82) | 0.0896 | 43.48 (20.65) | 0.0363 | 43.52 (20.76) | 0.0371 | 37.52(21.41) | 0.0812 |
| Vitamin D (nmol/L) | 0.03 (0.06) | 0.5569 | 0.02 (0.06) | 0.7197 | 0.03 (0.06) | 0.6433 | 0.03 (0.06) | 0.6624 |
| Mercury (nmol/L) | -0.008 (0.02) | 0.7073 | 0.001 (0.02) | 0.9562 | 0.0006 (0.02) | 0.9768 | -0.005 (0.02) | 0.8286 |
| Selenium (μmol/L) | -1.50 (2.48) | 0.5463 | 0.197 (2.33) | 0.9328 | 0.22 (2.35) | 0.9243 | -0.07 (2.43) | 0.9775 |
| Menopausal status | -1.13 (3.16) | 0.7220 | -1.93 (3.09) | 0.5330 | -1.75 (3.09) | 0.5730 | -1.95 (3.24) | 0.5480 |
| Level of education | 6.62 (2.46) | 0.0078 | 5.32 (2.43) | 0.0297 | 5.40 (2.44) | 0.0281 | 5.51 (2.61) | 0.0359 |
| Smoking status | -1.99 (2.14) | 0.3535 | -1.71 (2.11) | 0.4197 | -1.84 (2.12) | 0.3863 | -1.98 (2.25) | 0.3801 |
| Total lipid^(a)^ (g/L) | -0.24 (0.81) | 0.7625 | 0.06 (0.80) | 0.9438 | -0.03 (0.80) | 0.9679 | 0.20 (0.85) | 0.8191 |

^a^ Final adjustment for the total plasma lipid concentration.

Note: In additional analysis conducted by logistic regression, using the median BUA value (117.16 dB/MHz), DL-PCB 105 (0.03 µg/L) and DL-PCB 118 (0.18 µg/L) as bound to describe the two categories "low" and "high" BUA and exposure to DL-PCB congeners, significant results obtained by multiple linear regression were not confirmed. The odds ratio (OR) of 0.98 for low BUA at high DL-PCB 105 exposure (95% CI: 0.76-1.27) and OR of 1.01 for low BUA at high DL-PCB 118 exposure (95% CI: 0.786-1.31) were not significant (the models were adjusted for all variables indicated in this table).
