# Supplementary material for: Dioxin-like compounds and bone quality in Cree women of Eastern James Bay (Canada): a cross-sectional study
Source: Environ Health. 2013 Jul 2;12:54. doi: 10.1186/1476-069X-12-54 (PMC3704868; doi:10.1186/1476-069X-12-54)
Supplement: Additional file 3 — Multivariate analysis: SI (log) models (II). [file 1476-069X-12-54-S3.docx]

**Additional file 3**

Multivariate analysis: SI (log) models (II).

| **SI (log) model** | **Main exposure variable** | | | | | | | |
| --- | --- | --- | --- | --- | --- | --- | --- | --- |
|  | **DLC** (pg TCDD-EQ/L) | | **DL-PCB 105** (μg/L) | | **DL-PCB 118** (μg/L) | | **DL-PCB 156** (μg/L) | |
| **N**  **R^2^ (Adjusted R^2^)** | 242  0.3957 (0.3613) |  | 242  0.4075 (0.3737) |  | 242  0.4061 (0.3722) |  | 217  0.4086 (0.3708) |  |
|  |  |  |  |  |  |  |  |  |
|  | **Regression coefficient (SE)^(a)^** | **p-value** | **Regression coefficient (SE)^(a)^** | **p-value** | **Regression coefficient (SE)^(a)^** | **p-value** | **Regression coefficient (SE)^(a)^** | **p-value** |
|  |  |  |  |  |  |  |  |  |
| **Variables** | **DLC**  0.05 (0.09) | 0.5702 | **DL-PCB 105**  -251.61 (114.12) | 0.0285 | **DL-PCB 118**  -40.25 (19.38) | 0.0390 | **DL-PCB 156**  -67.96 (45.85) | 0.1399 |
|  |  |  |  |  |  |  |  |  |
| Age (years) | -13.86 (2.63) | <.0001 | -12.39 (2.44) | <.0001 | -12.58 (2.43) | <.0001 | -12.61 (2.55) | <.0001 |
| Weight (kg) | 0.07 (0.76) | 0.9321 | 0.27 (0.75) | 0.7182 | 0.18 (0.74) | 0.8045 | -0.07 (0.82) | 0.9317 |
| Height (cm) | -1.92 (2.38) | 0.4201 | -2.79 (2.34) | 0.2338 | -2.60 (2.34) | 0.2658 | -1.61 (2.52) | 0.5243 |
| Number of children | 0.37 (5.14) | 0.9420 | 5.11 (5.14) | 0.3207 | 4.51 (5.08) | 0.3765 | 3.31 (5.14) | 0.5208 |
| Omega-3/omega-6 PUFAs | 466.13 (272.76) | 0.0888 | 569.13 (270.54) | 0.0365 | 574.90 (271.61) | 0.0354 | 520.03 (280.45) | 0.0651 |
| Vitamin D (nmol/L) | 0.57 (0.74) | 0.4433 | 0.40 (0.73) | 0.5896 | 0.46 (0.73) | 0.5279 | 0.86 (0.81) | 0.2912 |
| Mercury (nmol/L) | 0.01 (0.28) | 0.9717 | 0.13 (0.28) | 0.6451 | 0.13 (0.28) | 0.6489 | 0.12 (0.30) | 0.6927 |
| Selenium (μmol/L) | 1.74 (32.46 ) | 0.9574 | 23.49 (30.48) | 0.4417 | 24.79 (30.76) | 0.4211 | 14.75 (31.81) | 0.6434 |
| Menopausal status | -29.24 (41.40) | 0.4808 | -39.55 (40.50) | 0.3299 | -37.41 (40.50) | 0.3565 | -41.15 (42.44) | 0.3334 |
| Level of education | 96.39 (32.27) | 0.0031 | 79.70 (31.86) | 0.0131 | 79.91 (31.95) | 0.0131 | 80.29 (34.14) | 0.0196 |
| Smoking status | -42.93 (28.01) | 0.1267 | -39.35 (27.69) | 0.1567 | -40.96 (27.71) | 0.1407 | -39.27 (29.51) | 0.1848 |
| Total lipid^(b)^ (g/L) | 3.25 (10.57) | 0.7589 | 7.13 (10.53) | 0.4989 | 6.12 (10.50) | 0.5606 | 8.10 (11.17) | 0.4693 |

^a^ Regression coefficients and standard errors values are multiplied *10^3^;

^b^ Final adjustment for the total plasma lipid concentration.

Note: In additional analysis conducted by logistic regression, using the median SI value (91%), DL-PCB 105 (0.03 µg/L) and DL-PCB 118 (0.18 µg/L) as bound to describe the two categories "low" and "high" SI and exposure to DL-PCB congeners, significant results obtained by multiple linear regression were confirmed. Therefore, the odds ratio (OR) of 1.48 for low SI at high DL-PCB 105 exposure (95% CI: 1.14-1.91) and OR of 1.52 for low SI at high DL-PCB 118 exposure (95% CI: 1.18-1.96) were significant (the models were adjusted for all variables indicated in this table).
