# Supplementary material for: Dioxin-like compounds and bone quality in Cree women of Eastern James Bay (Canada): a cross-sectional study
Source: Environ Health. 2013 Jul 2;12:54. doi: 10.1186/1476-069X-12-54 (PMC3704868; doi:10.1186/1476-069X-12-54)
Supplement: Additional file 4 — Other characteristics of participants. [file 1476-069X-12-54-S4.docx]

**Additional file 4**

Other characteristics of participants.

| **Characteristic** | **N** | **AM^(a)^ ± SD^(b)^** | **Range^(c)^** | **GM^(d)^ (95% CI)^(e)^** |
| --- | --- | --- | --- | --- |
|  |  |  |  |  |
| Vitamin E (μmol/L) | 249 | 30.23 ± 22.01 | 16.61-75.93 |  |
| Omega-3/omega-6 PUFAs | 248 | 0.23 ± 0.06 | 0.10-0.49 |  |
| Cadmium (nmol/L) | 249 | 11.80 ± 12.79 | 0.90-67.62 | 7.28 (6.45-8.22) |
|  |  |  |  |  |
|  | **N** | **N weighted (%)** |  |  |
| Parity  Yes  No | 247  209  38 | 2026.74  1748.05 (86.25)  278.69 (13.75) |  |  |
| Personal fracture history  Yes  No | 232  4  228 | 1881.49  31.57 (1.68)  1849.93 (98.32) |  |  |
| CSO^(f)^  Yes  No | 232  38  194 | 1881.49  274.93 (14.61)  1606.56 (85.39) |  |  |
| HRT use^(g)^  Yes  No | 249  2  247 | 2056.13  20.96 (1.02)  2035.17 (98.98) |  |  |
| Hormonal contraceptives use  Yes  No | 248  37  211 | 2042.25  299.66 (14.67)  1742.58 (85.33) |  |  |
| Alcohol intake  Yes  No | 248  68  180 | 2050.93  512.81 (25.00)  1538.12 (75.00) |  |  |
| Milk intake  Yes  No | 249  154  95 | 2056.13  1309.57 (63.69)  746.56 (36.31) |  |  |

^a^ Arithmetic mean (unweighted);

^b^ Standard deviation;

^c^ Minimum-maximum;

^d^ Geometric mean (unweighted);

^e^ 95% confidence interval of geometric mean;

^f^ Causes of secondary osteoporosis;

^g^ Hormone replacement therapy.

Note: These variables together with those given in Table 1 were part of the initial multivariate models before confounding analysis.
