# Supplementary material for: Dioxin-like compounds and bone quality in Cree women of Eastern James Bay (Canada): a cross-sectional study
Source: Environ Health. 2013 Jul 2;12:54. doi: 10.1186/1476-069X-12-54 (PMC3704868; doi:10.1186/1476-069X-12-54)
Supplement: Additional file 5 — Pearson’s correlation coefficients between age, plasma concentrations of DLC and DL-PCBs, and QUS parameters in Cree women. [file 1476-069X-12-54-S5.docx]

**Additional file 5**

Pearson’s correlation coefficients between age, plasma concentrations of DLC and DL-PCBs, and QUS parameters in Cree women.

|  | **SOS**  **(log, m/s)** | **BUA**  **(dB/MHz)** | **SI**  **(log, %)** | **DLC**  **(pg TCDD-EQ/L)** | **DL-PCB 105**  **(μg/L)** | **DL-PCB 118**  **(μg/L)** | **DL-PCB 156**  **(μg/L)** |
| --- | --- | --- | --- | --- | --- | --- | --- |
| **Variable** | **r^(a)^**  **p-value**  **N** | **r^(a)^**  **p-value**  **N** | **r^(a)^**  **p-value**  **N** | **r^(a)^**  **p-value**  **N** | **r^(a)^**  **p-value**  **N** | **r^(a)^**  **p-value**  **N** | **r^(a)^**  **p-value**  **N** |
|  |  |  |  |  |  |  |  |
| BUA (dB/MHz) | 0.75 <0.0001 249 |  |  |  |  |  |  |
|  |  |  |  |  |  |  |  |
| SI (log, %) | 0.92 <0.0001 249 | 0.93 <0.0001 249 |  |  |  |  |  |
|  |  |  |  |  |  |  |  |
| DLC (pg TCDD-EQ/L) | -0.30 <0.0001 249 | -0.34 <0.0001 249 | -0.36 <0.0001 249 |  |  |  |  |
|  |  |  |  |  |  |  |  |
| DL-PCB 105 (μg/L) | -0.28 <0.0001 249 | -0.37 <0.0001 249 | -0.38 <0.0001 249 | 0.75 <0.0001 249 |  |  |  |
|  |  |  |  |  |  |  |  |
| DL-PCB 118 (μg/L) | -0.29 <0.0001 249 | -0.37 <0.0001 249 | -0.38 <0.0001 249 | 0.75 <0.0001 249 | 0.98 <0.0001 249 |  |  |
|  |  |  |  |  |  |  |  |
| DL-PCB 156 (μg/L) | -0.31 <0.0001 223 | -0.40 <0.0001 223 | -0.40 <0.0001 223 | 0.73 <0.0001 223 | 0.84 <0.0001 223 | 0.90 <0.0001 223 |  |
|  |  |  |  |  |  |  |  |
| Age (years) | -0.50  <0.0001  249 | -0.56  <0.0001  249 | -0.59  <0.0001  249 | 0.66  <0.0001  249 | 0.54  <0.0001  249 | 0.55  <0.0001  249 | 0.60  <0.0001  223 |

^a^ Weighted values.
